# Pharmacokinetic-pharmacodynamic modeling to evaluate the relative impact of immune response and meropenem on bacterial killing *in vivo*

**DOI:** 10.1128/aac.01788-25

**Published:** 2026-02-20

**Authors:** Raphaël Saporta, Natália Tassi, Veronica Biordi, Olga Ticha, Aghavni Ginosyan, Irena Loryan, Elisabet I. Nielsen, Isabelle Bekeredjian-Ding, Bernhard Kerscher, Lena E. Friberg

**Affiliations:** 1 Department of Pharmacy, Uppsala University 8097, Uppsala, Sweden; 2 Division of Infectious Diseases, Paul-Ehrlich-Institut 39053, Langen, Germany; Providence Portland Medical Center, Portland, Oregon, USA

**Keywords:** meropenem, immune response, modeling, pharmacokinetics-pharmacodynamics

## Abstract

*In vivo* antibiotic pharmacokinetic-pharmacodynamic (PKPD) properties are typically studied in neutropenic infection models, limiting the understanding of interactions between immune cells and antibiotics. This study aimed to characterize the impact of immune status on meropenem PKPD and dose-response in a mouse lung infection model, and to quantify the relative contribution of immune response and meropenem to bacterial killing. Meropenem PK was analyzed in plasma and epithelial lung fluid, and bacterial counts were monitored over 24 h following 40 or 300 mg/kg meropenem doses every 4 h in a mouse lung infection model with varying immunosuppression levels (neutropenic, intermediate, or immunocompetent). A PKPD model was developed to quantify bacterial killing by the immune response and meropenem over time. Dose-fractionation studies were simulated to investigate meropenem dose-response in different immune states. Observed differences in meropenem concentration-time profiles were explained by a higher volume of distribution in immunocompetent mice. The immune response was described by phagocytosis and digestion processes. The meropenem effect was best quantified based on plasma concentrations, with a maximal killing rate of 0.934 h^−1^ and EC_50_ = 1.62 mg/L. The lower contribution of meropenem to bacterial killing in intermediate and immunocompetent conditions was explained as a lower fraction of bacteria being affected by meropenem. In simulations, the immune status impacted PKPD target derivation. The developed model characterized differences in meropenem PK between neutropenic and immunocompetent *in vivo* infection models and quantified the contributions of immune response and meropenem to bacterial killing, with a reduced effect of meropenem in immunocompetent systems.

## INTRODUCTION

Despite the importance of the immune response in infection control, its quantitative contribution to bacterial killing, particularly in the presence of antibiotics, is not fully understood. The innate immune response involves mechanisms to control and eliminate pathogens (1). Neutrophils, the most abundant circulating white blood cells in humans, employ different processes to induce bacterial killing, such as the phagocytosis of bacteria followed by the release of antibacterial proteins into the formed phagosome, degranulation, or the formation of neutrophil extracellular traps (2). The critical role of neutrophils is evidenced in patients with neutropenia (an absolute neutrophil count below 1.5 × 10^9^/L (3)), which is associated with an increased infection risk and mortality (4).

Although the immune response is crucial, it may not always be sufficient. Antibiotics, such as the carbapenem meropenem, are essential in the treatment of bacterial infections in clinical practice. During antibiotic drug development and regulatory evaluation, animal infection models are key in the pharmacokinetic-pharmacodynamic (PKPD) characterization (5, 6), with the neutropenic mouse thigh and lung infection models being most common (7). Neutropenia, usually induced by administering cyclophosphamide, allows sustained immunosuppression over the study duration (8, 9). The antibiotic PKPD is, therefore, characterized under conditions that limit the confounding of drug effects with immune response. Additionally, more strains can successfully grow in neutropenic conditions. However, varying degrees of immune response are expected in clinical practice, yet these preclinical models neither quantify immune-related bacterial killing nor evaluate potential interplays between the immune system and the antibiotic’s PKPD properties.

PKPD modeling has been increasingly employed to characterize the time course of antibiotic effects, based on *in vitro* and/or *in vivo* data (10–12). However, few studies have quantified the impact of immunosuppression on bacterial killing (13–16), and models addressing both drug effect and immune response are particularly scarce (17, 18).

The PKPD of meropenem has been extensively studied under both *in vitro* and neutropenic *in vivo* conditions, including through PKPD modeling (19, 20). However, despite the importance of carbapenems in clinical practice (21), little is known about how the PKPD of meropenem is dependent on immunocompetent conditions. This study aimed to characterize meropenem PK and PKPD in neutropenic and immunocompetent conditions by quantifying the relative contributions of meropenem and the immune system to bacterial killing in a mouse lung infection model using PKPD modeling, and to explore the impact of the immune system on meropenem dose-response and derived PKPD targets through simulations.

## MATERIALS AND METHODS

### Mouse lung infection model

Specific pathogen-free CD-1 female mice (Janvier Labs, France) of 6–10 weeks of age at intervention start were used, in line with a standardized murine lung infection model, the COMBINE pneumonia model (9), with modifications to the standard model regarding the immune status. Two different intraperitoneal dosing regimens of cyclophosphamide (Baxter, Germany) were used to induce different states of immunosuppression in mice: the intermediate state was obtained with 75 and 50 mg/kg intraperitoneal doses at 4 and 1 days before infection, respectively, and the neutropenic state with 200 and 150 mg/kg intraperitoneal doses at the same time points. For the immunocompetent state, no cyclophosphamide was administered.

*Klebsiella pneumoniae* DSM116099 was obtained from the German culture collection DSMZ (Leibniz Institute, Germany). Meropenem minimum inhibitory concentration (MIC) was identified at 0.032 mg/L by determination in triplicate by microdilution broth according to the EUCAST guidelines (22). On the day of infection, bacteria were inoculated into fresh tryptic soy broth media from an overnight culture, grown to logarithmic phase, washed with phosphate-buffered saline (PBS) twice, and resuspended in PBS to the desired concentration, based on individually established correlation curves. Mice were anesthetized with 3.5% isoflurane in oxygen-enriched air, and bacterial suspensions were applied intranasally by depositing 50 µL of suspension into the nares and allowing the mice to inhale.

Meropenem (Inresa Arzneimittel GmbH, Germany) was administered subcutaneously based on individual animal weights. For PK experiments, a single dose of 40 or 300 mg/kg was given at 2 h post-infection. For PD experiments, doses of 40 or 300 mg/kg were given every 4 h, starting at 2 h post-infection.

Mice were euthanized by CO_2_ or anesthetics overdose followed by cervical dislocation when reaching the humane endpoint/severity limit or at the study endpoint. In PK (*n* = 60 mice) and PD (*n* = 180 mice) experiments, blood, bronchoalveolar lavage (BAL), and whole lung tissue samples were taken at times detailed in Table S1.

### Sampling and analyses

In PK experiments, plasma samples were collected in EDTA tubes from superficial veins or by cardiac puncture under terminal FDA ketamine-xylazine anesthesia. Total meropenem concentration in plasma and BAL samples was quantified using Acquity ultra-performance liquid chromatography coupled with Xevo TQ-S Micro triple quadrupole mass spectrometer (UPLC-MS/MS, Waters Corporation, Milford, MA, USA). Data acquisition and quantitation were done using Masslynx v4.2 (Waters Corporation). The method for the assessment of plasma concentrations was developed based on previously published protocols with modifications (23–25). The bioanalytical method was validated for selectivity, response function, range, intra- and inter-day accuracy, precision, matrix effect, and carryover according to the FDA guidance (26). The lowest standard concentration (0.05 mg/L) was set as the lower limit of quantification (LLOQ) in plasma and BAL. Meropenem concentrations in epithelial lining fluid (ELF) were derived from meropenem concentrations in BAL by urea correction (*C*_ELF_ = *C*_BAL_ × Urea_plasma_/Urea_BAL_).

In PD experiments, general blood cell counts were determined by a veterinary clinical blood analyzer (scil animal care company GmbH, Germany). For CFU enumeration, right lungs were collected in Lysing Matrix D (MP Biomedicals, Germany) containing 1 mL of 0.1% Triton X-100 (Sigma-Aldrich, Germany) in PBS. The tissue homogenate was generated by three cycles of bead-beating (5,000 rpm, 13 s/cycle with 15-s breaks) on a Pracellys 24 touch (Bertin Technologies, Germany). Serial dilutions in PBS were prepared in duplicate. Dilutions 10^−1^ to 10^−8^ were plated on tryptic soy agar and incubated at 37°C overnight.

### PK modeling

Compartmental models were investigated to describe meropenem plasma and lung (ELF) concentrations. Absorption following subcutaneous (SC) drug administration was evaluated as a first- or zero-order process. Potential differences in PK parameters between immunocompetent and neutropenic mice were assessed.

### PKPD modeling

The PKPD model structure was developed in two steps. First, data from control groups (without antibiotic treatment) were analyzed alone to develop the model structure for bacterial growth and immune response. Second, data from treatment groups were incorporated, and all data were analyzed simultaneously to characterize the meropenem effect.

Bacteria were described to be in a growing and drug-susceptible (S) state, or in a dormant (or resting), non-growing and non-drug-susceptible (D) state (27). Bacterial growth was defined by a rate constant *k*_growth_. In both states, a non-immune-related natural death rate constant *k*_death_ was implemented. The rate constant for transfer from S to D, *k*_SD_, was linked to the current total bacterial count and the maximum system capacity, *B*_max_, with *k*_SD_ = (*S* + *D*)∙(*k*_growth_ − *k*_death_)/*B*_max_. For each immune state, the S compartment was initialized to the median bacterial count from control groups at 2 h after infection. The immune response was described as a phagocytosis and digestion process (16). Bacteria in the S and D states transferred to a third bacterial state, phagocytosis (P), at a rate *k*_phag_, followed by their digestion and removal from the system at a rate *k*_dig_. Bacteria in the P state were also assumed to contribute to the total CFU counts but not to *B*_max_, as phagocytic cells were lysed prior to the counting procedure.

The rates *k*_phag_ and *k*_dig_ were assessed to be dependent on immune status, either as condition-specific parameters or by assuming they were related to blood or lung granulocyte counts. Granulocyte recruitment to the infection site was investigated either through a function for a time-dependent increase in *k*_phag_, an effect compartment, or a surge function for granulocyte recruitment to the lungs using previously estimated parameters (16). As the killing rate attributable to granulocytes was previously shown to be subject to saturation at high bacterial burdens (14–16), functions were explored to describe a reduced phagocytic activity with increasing P bacteria compared to the number of available granulocytes.

The meropenem effect was implemented as an additional bacterial killing rate, *k*_drug_, on bacteria in the S state. Bacterial dynamics were described by the following equations:


(1) $$\begin{array}{c} \frac{\text{dS}}{\text{dt}}=k_{\text{growth}}\cdot S-\left(k_{\text{death}}\text{+}k_{\text{drug}}\right)\cdot S-k_{\text{SD}}\cdot S-k_{\text{phag}}\cdot S \end{array}$$

(2) $$\begin{array}{c} \frac{\text{dD}}{\text{dt}}=k_{\text{SD}}\cdot S-k_{\text{death}}\cdot D-k_{\text{phag}}\cdot D \end{array}$$

(3) $$\begin{array}{c} \frac{\text{dP}}{\text{dt}}=k_{\text{phag}}\cdot S+k_{\text{phag}}\cdot D-k_{\text{dig}}\cdot P \end{array}$$

Unbound meropenem plasma (fu = 0.81) (19) or ELF concentrations predicted from the PK model were used to drive the drug effect. Meropenem effect was evaluated with slope or power (*γ* = 1 or *γ* ≠ 1, equation 4), *E*_max_ or sigmoid *E*_max_ (*γ* = 1 or γ ≠ 1, equation 5) models:


(4) $$k_{drug}=Slope\cdot C^{\;\gamma }$$

(5) $$\begin{array}{c} k_{\text{drug}}=\frac{E_{\max}\cdot C^{\gamma }}{{\text{EC}}_{\text{50}}^{\gamma }+C^{\gamma }} \end{array}$$

where *C* is the unbound meropenem concentration, Slope is the drug effect slope, *E*_max_ is the maximum drug effect, EC_50_ is the meropenem concentration at which 50% of *E*_max_ is reached, and *γ* is the power or sigmoidicity parameter. Drug effect parameters were estimated simultaneously with other PD parameters. The possibility that meropenem affects P bacteria was also evaluated.

### Simulation of dose-ranging and dose-fractionation studies

The final PKPD model was used to predict response in meropenem studies in a mouse lung infection model in neutropenic, intermediate suppression, and immunocompetent conditions. All simulations were performed considering an inoculum of 7.5 log_10_ CFU/lung, and without residual variability. A dose-ranging study with doses ranging from 20 to 1,200 mg/kg administered every 4 h, and a dose-fractionation study with total doses equivalent to those of the 40 and 300 mg/kg every 4 h regimens, fractionated in administrations every 2, 4, 6, or 8 h, were simulated to explore the predicted bacterial dynamics over 24 h of treatment. Additionally, a dose-fractionation study was simulated based on a literature design (28), with a start of treatment 2 h after inoculation. Simulated bacterial counts at 26 h (24 h after start of treatment) were correlated to derived PK/PD indices: the unbound maximum concentration (*fC*_max_) to MIC ratio (*fC*_max_/MIC), the area under the unbound concentration-time profile curve (*f*AUC) to MIC ratio (*f*AUC/MIC), and the fraction of time unbound concentrations exceeded the MIC (*fT*_>MIC_). The relationship between 26-h bacterial counts and PK/PD indices was evaluated with a sigmoid *E*_max_ function:


(6) $$\begin{array}{c} E=E_{\text{0}}-\frac{{\text{PD}}_{\max}\cdot {\text{Index}}^{H}}{{\text{Index}}_{\text{50}}^{H}+{\text{Index}}^{H}} \end{array}$$

where *E* is the 26-h bacterial load, *E*_0_ is the 26-h bacterial load when Index = 0, PD_max_ is the maximum reduction in bacterial load from *E*_0_ after 24 h of treatment, *H* is the Hill factor for sigmoidal shape, Index is the PK/PD index value, and Index_50_ is the PK/PD index value required to reach half of PD_max_. Correlations between simulated bacterial counts and fitted *E*_max_ curves were computed (*R*^2^ values).

### Data analysis and software

Model development was performed in NONMEM 7.5.0 (29), assisted by Perl-speaks-NONMEM version 5.2.6 (30), using the Laplacian conditional estimation method with interaction. PK modeling was performed using a log-transform-both-sides approach, and data below the LLOQ were handled using the M3 method (31). A log_10_-transform-both-sides approach was used for PKPD modeling. Residual errors were described by additive terms on the log-transformed scales. The difference in the objective function value (ΔOFV) was used to evaluate parameter inclusion, with a decrease in OFV of 3.84 points considered statistically significant for the inclusion of one extra parameter (*α* = 0.05). Other model selection criteria were scientific plausibility, parameter uncertainty as estimated using sampling importance resampling (32), and goodness-of-fit plots including simulation-based visual predictive checks (VPCs) (33). Data management and graphical analysis were conducted in R version 4.3.3 (34). Simulations were carried out in NONMEM 7.5.0 and in R using mrgsolve version 1.5.1 (35). Curve fitting for PK/PD indices was performed using drc version 3.0-1 in R (36).

## RESULTS

### Mouse PK experiments and PK modeling

**Fig 1:**
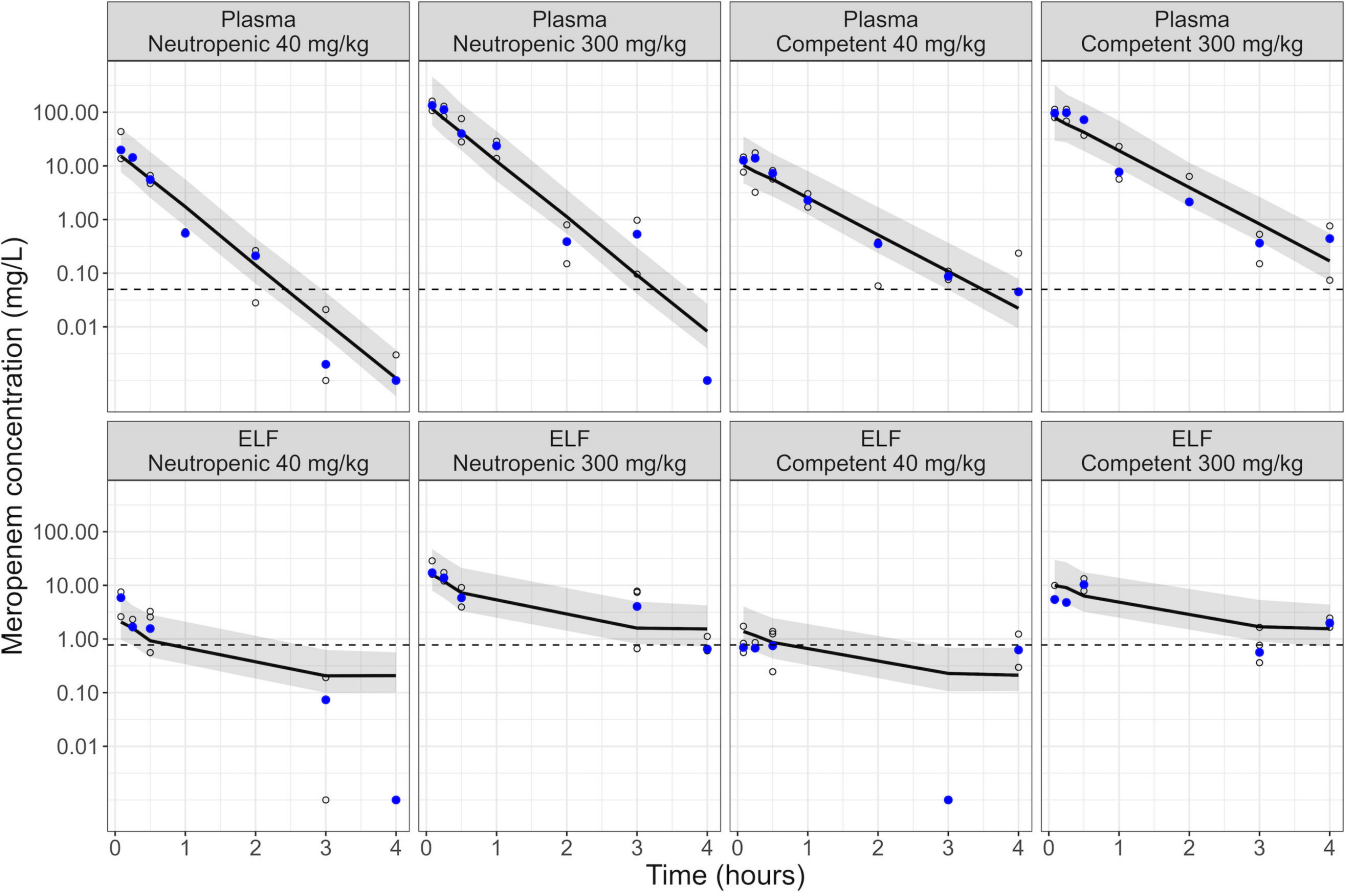
VPCs of the final PK model. Shown are the observed (open circles) and median of observed (solid blue circles) meropenem concentrations, the median (solid line) and the 95% confidence interval of the median (shaded area) of model predictions in plasma and ELF for the meropenem doses in neutropenic and immunocompetent mice. The lower limit of quantification is indicated by the horizontal dashed line, with the lower limit of quantification in ELF derived based on urea correction from the limit of quantification in BAL. Observations below the limit of quantification are displayed at their measured (plasma) or derived (ELF) value.

Meropenem plasma PK following SC administration was captured by a one-compartment model, with first-order absorption, while lung concentrations in ELF were described by a two-compartment model. Mass transfer between plasma and lung compartments was not retained in the model to improve stability and provide reliable plasma predictions. Differences in plasma and ELF concentration-time profiles were observed between neutropenic and immunocompetent mice, best described by different central volumes of distribution, with a central volume of 2.19 and 3.40 L/kg for neutropenic and immunocompetent mice, respectively (ΔOFV = −29.4 compared to a model in which the same volume of distribution was shared between both immune states). In the PKPD modeling, the meropenem central volume of distribution in mice with intermediate suppression was scaled to 2.46 L/kg by linear interpolation based on median white blood cell counts from the PD experiments. Parameters were estimated with acceptable precision (relative standard errors [RSE] < 40%, Table 1). VPCs suggested a good description of plasma and ELF concentrations for both dose groups and both immune states (Fig. 1).

**TABLE 1:** Parameter estimates and relative standard errors (RSEs) of the final model in neutropenic, intermediate suppression, or immunocompetent mice

Parameter	Unit	Description	Estimate	RSE (%)
CL	L/(h·kg)	Apparent clearance	5.33	11
*V*_neu_	L/kg	Apparent central volume of distribution (neutropenic)	2.19	18
*V*_int_	L/kg	Apparent central volume of distribution (intermediate suppression)	2.46^*a*^	Fixed
*V*_com_	L/kg	Apparent central volume of distribution (competent)	3.4	18
*k*_a_	h^−1^	Absorption rate constant	71.3	39
*V*_ELF_	L/kg	Apparent ELF volume of distribution	0.0554	23
*V*_L2_	L/kg	Apparent volume of distribution of second lung compartment	9.27	26
*Q*	L/(h·kg)	Apparent intercompartmental clearance (central-ELF)	0.385	19
*Q*_2_	L/(h·kg)	Apparent intercompartmental clearance (ELF-lung)	2.48	13
*k*_growth_	h^−1^	Bacterial growth rate constant	0.372	11
*k*_death_	h^−1^	Bacterial natural death rate constant	0.179^*a*^	Fixed
*B*_max_	log_10_ CFU/lung	Maximum bacterial count	9.59^*a*^	Fixed
IR_neu_	h^−1^	Phagocytosis rate (*k*_phag_) in neutropenic state *k*_phag_ = IR_neu_	0^*a*^	Fixed
IR_int_	h^−1^	Increase in phagocytosis rate in intermediate suppression state *k*_phag_ = IR_neu_ + IR_int_	0.185	12
IR_com_	h^−1^	Increase in phagocytosis rate in immunocompetent state *k*_phag_= IR_neu_ + IR_int_ + IR_com_	0.133	18
*E*_max_	h^−1^	Maximum meropenem effect (killing rate constant)	0.934	14
EC_50_	mg/L	Concentration needed to achieve 50% of *E*_max_	1.62	18
RES_Plasma_	–	Residual error of ELF PK (standard deviation)	0.816	17
RES_ELF_	–	Residual error of plasma PK (standard deviation)	0.738	22
RES_PD_	log_10_ CFU/lung	Residual error of the PKPD model (standard deviation)	0.623	14

^*a*^ Fixed parameters.

### Mouse PD experiments and PKPD modeling

**Fig 2:**
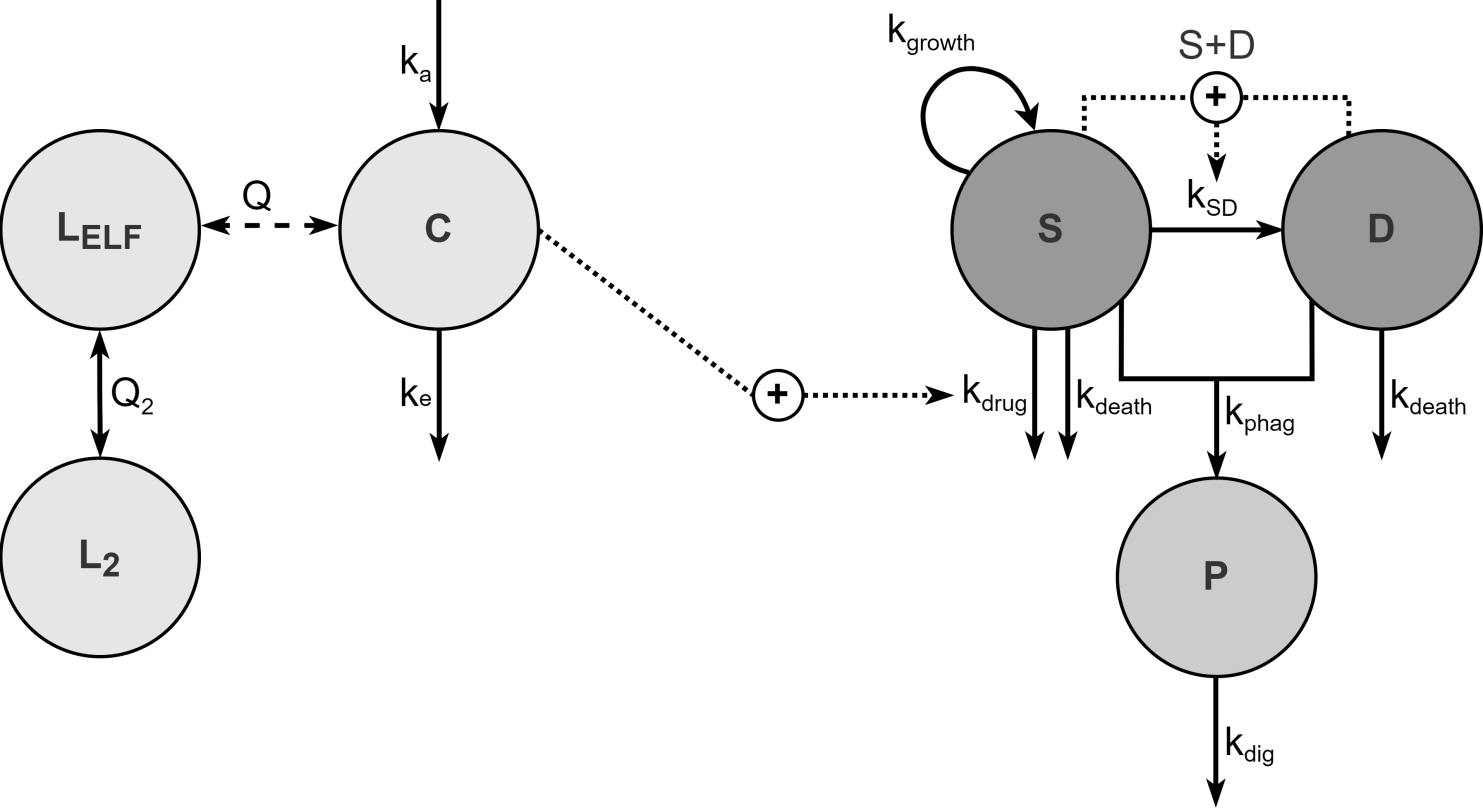
Schematic representation of the final PKPD model. C, meropenem plasma concentration. D, dormant bacteria. k_a_, absorption rate constant. k_death_, bacterial natural death rate constant. k_dig_, digestion rate constant. k_drug_, meropenem effect rate constant. k_e_, elimination rate constant. k_growth_, bacterial growth rate constant. k_phag_, phagocytosis rate constant. k_SD_, transfer from susceptible to dormant state rate constant. L_ELF_, meropenem ELF compartment. L_2_, meropenem second lung compartment. P, phagocytosed bacteria. S, susceptible bacteria. Q and Q_2_, intercompartmental clearances.

Median observed bacterial counts at 2 h after infection were 7.56, 7.80, and 7.52 log_10_ CFU/lung for neutropenic, intermediate, and competent groups, respectively. Small to no time trends were observed in white blood cell and blood granulocyte counts (Fig. S1 and S2, observed cell type distribution presented in Table S2. Consequently, modeling of granulocyte blood dynamics was not explored, but median granulocyte counts (0.2 × 10^3^, 0.4 × 10^3^, and 1.9 × 10^3^/mm^3^ in neutropenic, intermediate, and immunocompetent states, respectively) were investigated to scale *k*_phag_ and/or *k*_dig_. This led to a worse model fit compared to using the immune status as a categorical covariate. The structure of the final model is presented in Fig. 2, with parameter estimates and associated uncertainties in Table 1. As no bacterial plateau phase was visible in control groups, *B*_max_ could not be reliably estimated (high standard errors and/or non-plausible estimates) and was, thus, fixed to the median bacterial count in neutropenic controls at 26 hours after infection. A saturation of the immune response, or functions describing the recruitment of granulocytes to the infection site, was not supported by the data. The immune response was explored using parameters for each immune state. The phagocytosis rate in neutropenic conditions, IR_neu_, was not differentiable from *k*_growth_, and was therefore fixed to 0, while *k*_growth_ was estimated at 0.372 h^−1^. The phagocytosis rate in intermediate and immunocompetent conditions was estimated at 0.185 and 0.318 h^−1^, respectively. The digestion rate *k*_dig_ was not significantly different from *k*_phag_.

**Fig 3:**
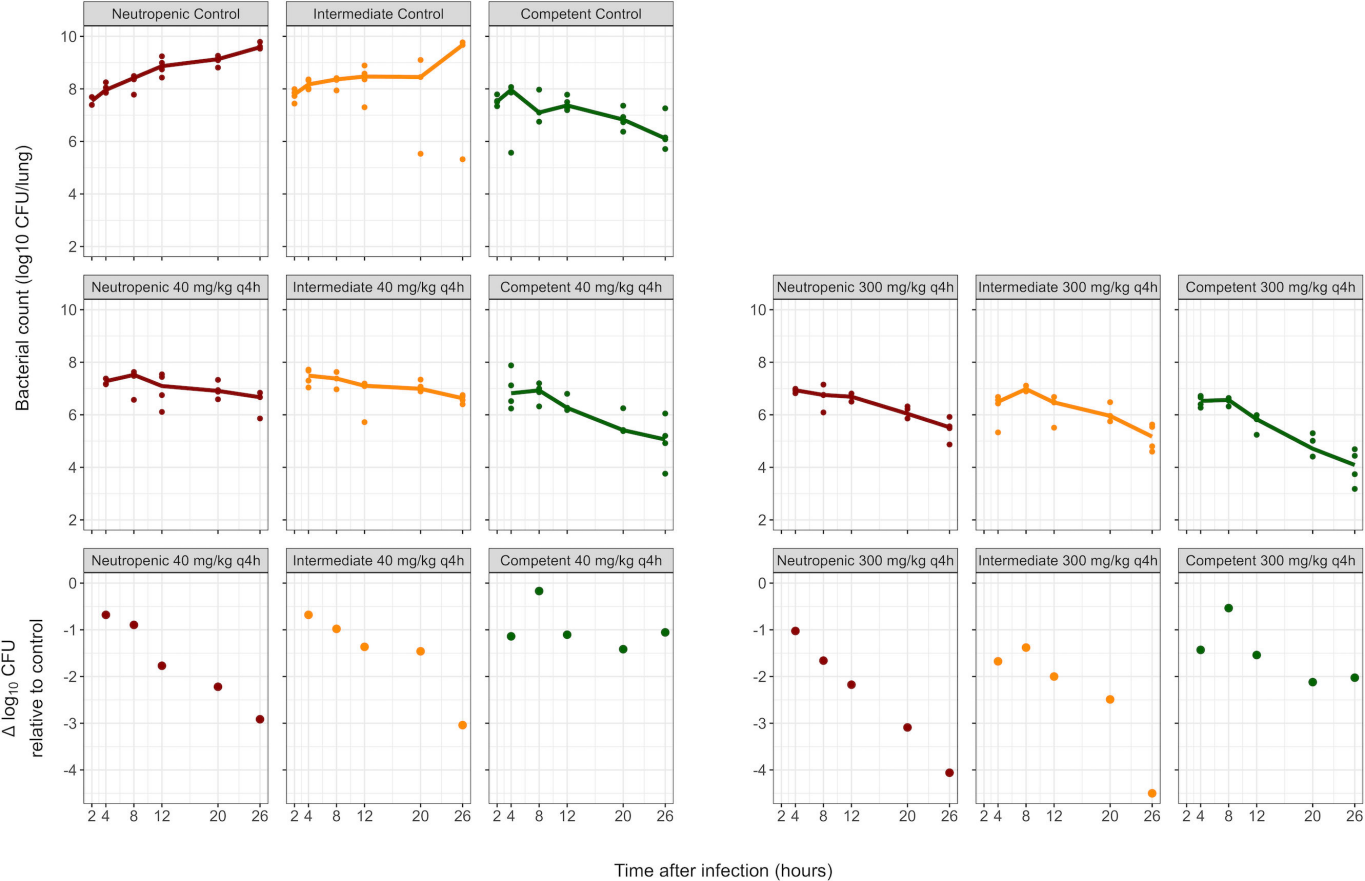
Bacterial counts measured in the PD experiments in neutropenic, intermediate suppression, and immunocompetent mice. Shown are observed (solid circles) and medians (solid lines) of bacterial counts in control groups (top row) and treatment groups (middle row). The bottom row depicts the differences in bacterial counts (in log_10_ CFU/lung) between median bacterial counts in treatment groups and median bacterial counts in control groups for the corresponding times and immune states.

**Fig 4:**
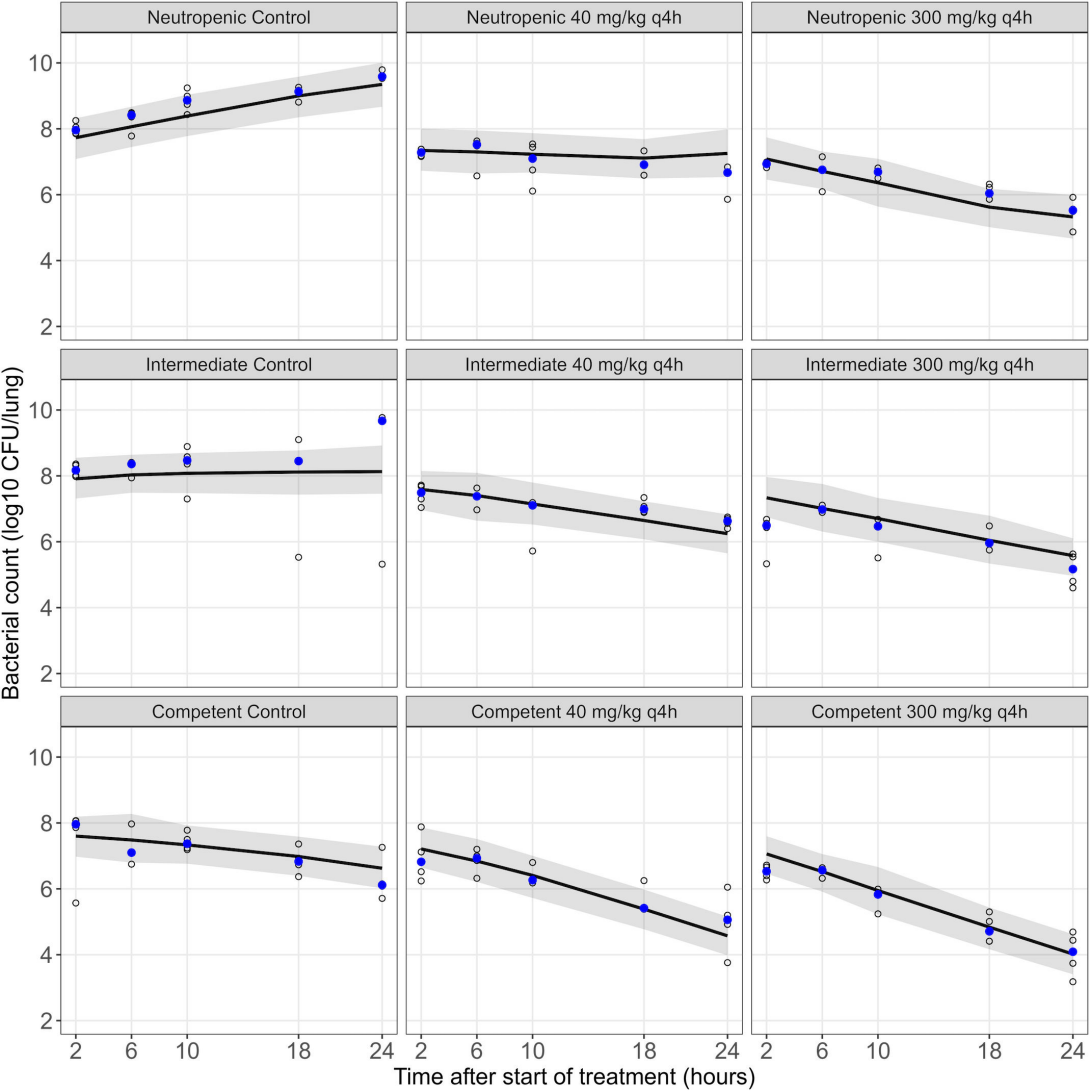
VPCs of the final PKPD model. Shown are the observed (open circles) and median of observed (solid blue circles) bacterial counts, the median (solid line) and 95% confidence interval of the median (shaded area) of model predictions for the meropenem dose groups in mice in a neutropenic, intermediate suppression, and immunocompetent state.

Bacterial counts (Fig. 3) were lower overall in control and treatment groups for immunocompetent conditions. However, the contribution of meropenem to bacterial killing was dependent on the immune status, with reduced meropenem-related bacterial killing in intermediate and immunocompetent groups. For example, the median decrease in CFU at 26 h in the 300 mg/kg q4h group compared to controls was ~4 log_10_ CFU in neutropenic mice versus ~2 log_10_ CFU in immunocompetent mice. The model fit was better (ΔOFV = −15.7) when the plasma concentration-time profile, rather than the ELF concentration-time profile, was driving the bacterial killing due to meropenem. Power and *E*_max_ models showed similar performances (ΔOFV = −1.8 for the *E*_max_ model). The *E*_max_ model was selected (Table 1) for biological plausibility and interpretability. The final model could capture observed bacterial counts across dose groups and immune states (Fig. 4).

### Simulated dose-ranging and dose-fractionation studies

**Fig 5:**
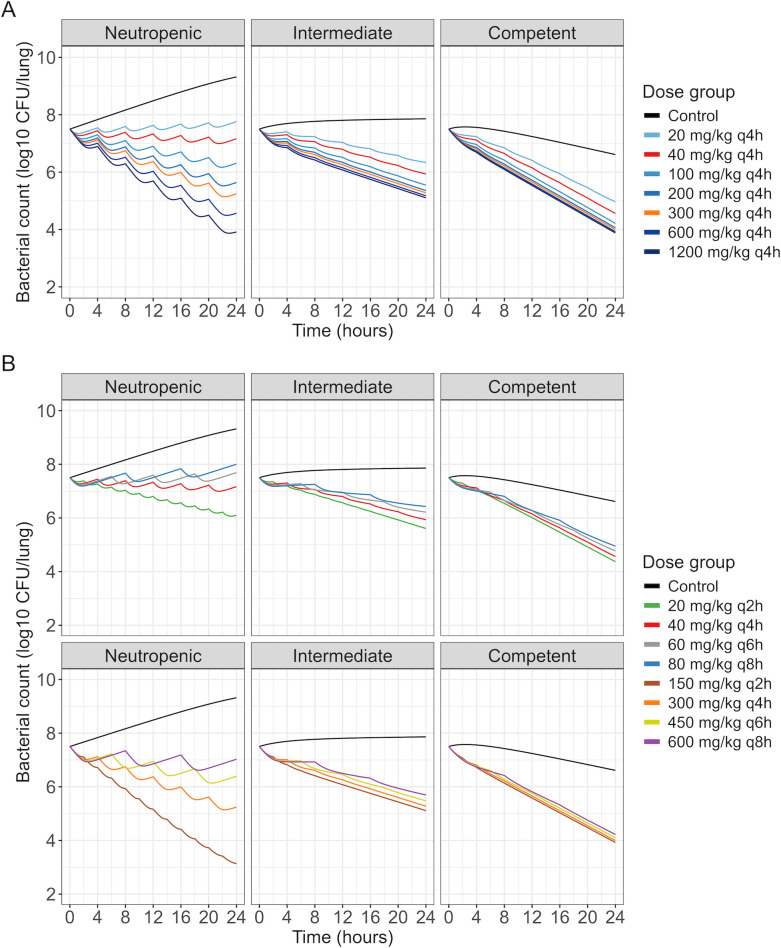
Predicted bacterial counts in mice in a neutropenic, intermediate suppression, or immunocompetent state based on the final PKPD model for (**A**) a dose-ranging study of meropenem and (**B**) a dose-fractionation study of meropenem (top row: fractionation of the first total dose, bottom row: fractionation of the second total dose).

As in the original data, a larger relative difference in 24-h bacterial count between doses was predicted in neutropenic mice compared to the other immune states (Fig. 5), indicating a reduced dose-response gradient in immunocompetent conditions (Fig. S3). Similarly, in dose-fractionation studies, although frequent administrations led to the lowest bacterial counts in all immune states, the difference in predicted bacterial killing between q2h administrations and other administration intervals was greater in neutropenic mice. The lowest doses or longest dosing intervals (e.g., 20 mg/kg q4h and 80 mg/kg q8h), predicted to achieve growth or stasis in neutropenic mice, achieved net killing in intermediate and immunocompetent mice at 24 h. For the highest or most frequent doses (e.g. 1,200 mg/kg q4h and 150 mg/kg q2h), lower bacterial counts at 24 h were predicted in neutropenic mice compared to mice with intermediate suppression.

**Fig 6:**
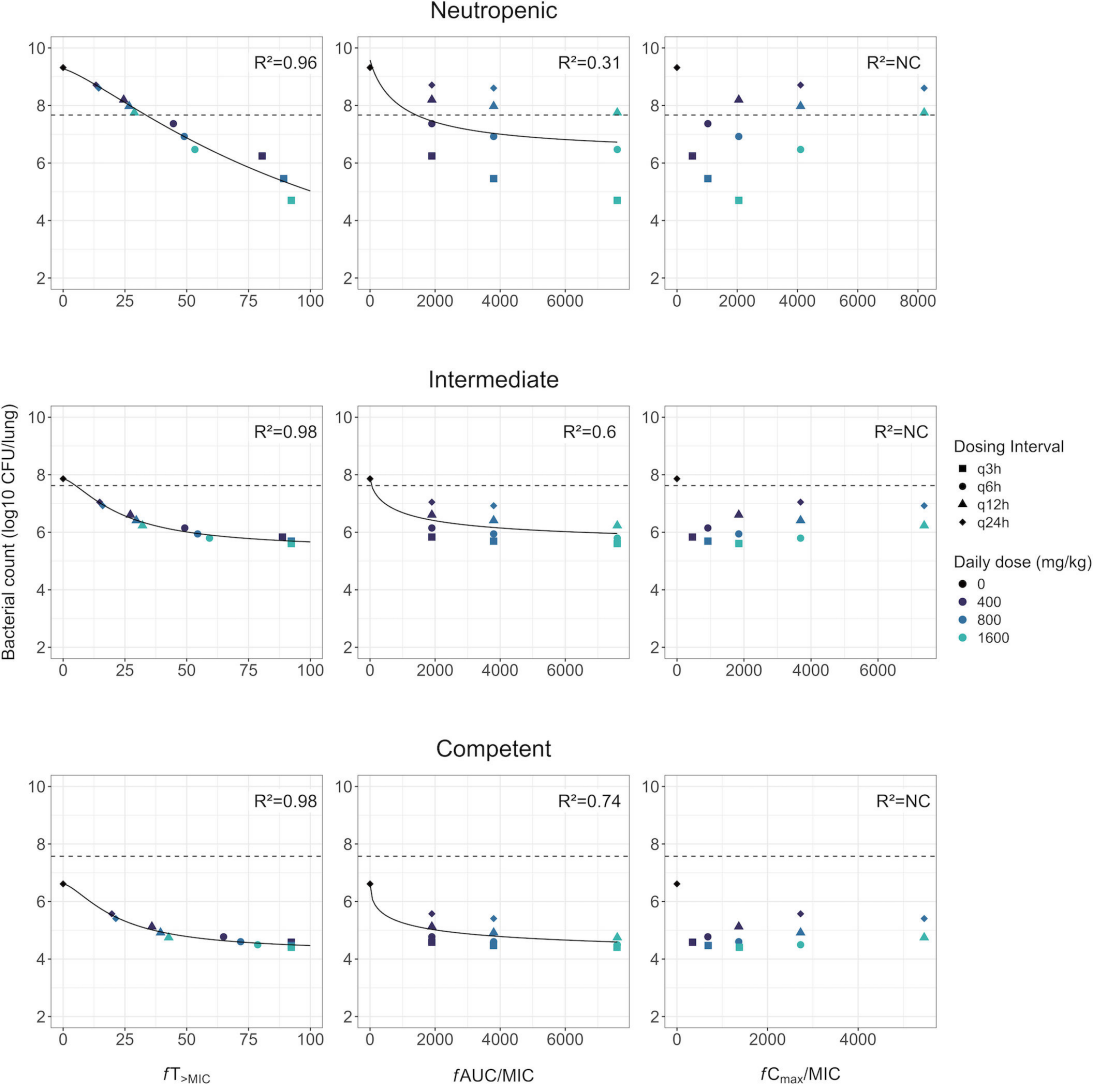
Relationship between plasma PK/PD indices and *K. pneumoniae* DSM116099 (MIC = 0.032 mg/L) bacterial counts at 26 h (24 h after start of treatment) in simulated meropenem dose-fractionation studies in mice in a neutropenic, intermediate immunosuppression, and immunocompetent state. Shown are the simulated bacterial counts (shapes), the stasis line (horizontal dashed line, corresponding to the bacterial count in control groups at 2 h after inoculation), the fitted *E*_max_ curve (solid line), and the *R*^2^ value reported in each panel. NC, not computed.

For all immune states, the PK/PD indices (Fig. 6) showed the highest correlations for *fT*_>MIC_ with predicted bacterial counts after 24 h of treatment (*R*^2^ > 0.9). The *R*^2^ value of *f*AUC/MIC was nonetheless noticeably improved in immunocompetent conditions (*R*^2^ = 0.74 and 0.31 in immunocompetent and neutropenic states, respectively). A near-maximal effect was reached at lower *fT*_>MIC_ values in intermediate and immunocompetent compared to neutropenic conditions. However, the log_10_ decrease in bacterial counts, compared to controls, at high *fT*_>MIC_ values was lower in immunocompetent compared to neutropenic conditions.

## DISCUSSION

This study used a standardized *in vivo* mouse lung infection model (9), with varying levels of immunosuppression, to quantify the immune response’s contribution to bacterial killing and its impact on the PKPD of meropenem over time via a PKPD model.

Observed meropenem plasma and ELF concentrations indicated different PK profiles depending on the immune status. Similar trends have earlier been observed for tedizolid in ELF (37). A reduced volume of distribution in plasma in immunosuppressed mice could be due to cyclophosphamide affecting general well-being and fluid balance, or due to less meropenem being able to penetrate into immune cells (38). These findings highlight the importance of assessing antibiotic PK in the experimental conditions to be used in PKPD analyses.

The developed PKPD model described the immune response with an estimate of the apparent bacterial growth (accounting for immune response) in neutropenic mice, and a phagocytosis rate of 0.185 and 0.318 h^−1^ in intermediate and competent mice. Granulocyte dynamics could not be described, as blood counts showed no increase over 24 h, consistent with the multiple days required for neutrophil recovery after cyclophosphamide treatment (8). Furthermore, no delay and variations in immune response related to the neutrophil recruitment to the infection site could be characterized in the PKPD model, as the time delay between the phagocytosis and digestion processes was sufficient to describe the observed data. In neutropenic conditions, the impact of the immune system was not differentiable from the bacterial growth rate. Additional data, such as experiments with different inoculum sizes, would be required to differentiate bacterial growth from the immune response in neutropenic conditions.

The contribution of meropenem to bacterial killing depended on the immune status, with a higher bacterial killing in neutropenic conditions compared to other immune states when comparing treatment and control groups. Similar behaviors were observed in the tedizolid dose-response in a neutropenic versus immunocompetent mouse thigh infection model (17). Nonetheless, there was no significant difference between immune states in the meropenem effect parameters. The observed differences were best explained by a higher proportion of the bacteria transferred to the P compartment in immunocompetent conditions, and thereby a lower fraction of the bacteria was exposed to meropenem in the S compartment. Consequently, although the internalized bacteria would eventually be cleared from the system, meropenem would be responsible for a lower reduction of CFU in immunocompetent conditions compared to neutropenic conditions. The data did not support an effect of meropenem within the immune cells. A lower intracellular activity of meropenem in monocytes, compared to extracellular conditions, has been shown *in vitro* (39).

In the simulated dose-ranging and fractionation studies, the dose-response gradient’s dependence on immune status was illustrated. Bacterial counts were predicted to be at least as low in neutropenic conditions as in immunocompetent conditions for the highest doses or the most frequent administration schedule, while doses predicted to achieve stasis or growth in neutropenic conditions were predicted to achieve killing in immunocompetent conditions (Fig. 5).

In dose-fractionation studies, frequent administrations were predicted to be the most effective for a given total dose, with *fT*_>MIC_ identified as the PK/PD index with the highest correlation, as previously reported for meropenem (28, 40). Nonetheless, differences in bacterial counts compared to other dosing schedules were less apparent in immunocompetent conditions, impacting the predicted antibiotic efficacy for PK/PD index values. A near-maximal reduction of bacterial counts was achieved at *fT*_>MIC_ ≈40% in immunocompetent mice, whereas an additional ~2 log decrease in counts was predicted when increasing *fT*_>MIC_ from 40% to 80% in neutropenic mice. Derived efficacy targets would thus be expected to differ if performing a dose-fractionation study in a neutropenic or immunocompetent setting. It should be noted that for the studied strain, as bacterial counts in immunocompetent control groups were predicted below the stasis line at 26 h, targets would need to be tailored to other criteria than in neutropenic conditions. Overall, these results showed that conclusions drawn for antibiotic efficacy and dose selection could differ between the neutropenic and immunocompetent mouse infection models. Studies in immunocompetent mice could potentially provide insights into differences in efficacy between patient populations with different immune states, as well as improve the understanding of how different antibiotics depend on the immune system. As a perspective, *in vivo* studies with different types of immune deficiencies (using SCID, NOD, or gammac mouse models) may further inform how antibiotics and the immune system interact.

The developed model had some limitations. It was not possible to quantify a saturation of the immune response (14–16) based on available data, which would likely require expanded studies using several bacterial inoculum sizes. Furthermore, the group-dependent phagocytosis rate, in contrast to a continuous function, limits the capacity to extrapolate to unstudied conditions. As a single bacterial strain was studied, further explorations with other bacterial strains or species would be required to enhance the model’s robustness.

In conclusion, the current study identified differences in meropenem PK between neutropenic and immunocompetent mouse infection models and quantified bacterial killing by the immune response and meropenem in a mouse lung infection model with varying degrees of immunosuppression. Meropenem was found to have a reduced contribution to bacterial killing in immunocompetent conditions, attributed to a lower fraction of bacteria affected by meropenem. Simulations demonstrated that outcomes of antibiotic PKPD analyses, such as PK/PD targets derived from PK/PD indices, will vary depending on the use of an immunocompetent or a neutropenic infection model.

## Data Availability

Data will be made available on request from the corresponding author.

## SUPPLEMENTAL MATERIAL

Supplemental material aac.01788-25-s0001.docx Tables S1 and S2; Fig. S1 to S3.

ASM does not own the copyrights to Supplemental Material that may be linked to, or accessed through, an article. The authors have granted ASM a non-exclusive, world-wide license to publish the Supplemental Material files. Please contact the corresponding author directly for reuse.
