# Supplementary material for: Pharmacokinetic-pharmacodynamic modeling to evaluate the relative impact of immune response and meropenem on bacterial killing in vivo
Source: Antimicrob Agents Chemother. 2026 Feb 20;70(4):e01788-25. doi: 10.1128/aac.01788-25 (PMC13041408; doi:10.1128/aac.01788-25)
Supplement: Supplemental material — Tables S1 and S2; Fig. S1 to S3. [file aac.01788-25-s0001.docx]

# Supplementary material

**Table S1.** Summary of samples taken in the pharmacokinetic (PK) and pharmacodynamic (PD) experiments

| Experiment | Number of mice | Samples | Samples per animal | Time points |
| --- | --- | --- | --- | --- |
| PK | 60 | Blood | 1 to 2 | 5, 15, 30, 60, 120, 180, 240 minutes after administration |
|  |  | Bronchoalveolar lavage (BAL) | 1 | 5, 15, 30, 180, 240  minutes after administration |
| PD | 180 | Blood | 1 | 2, 4, 8, 12, 20, 26  hours after infection |
|  |  | Whole lung tissue | 1 |  |

**Table S2.** Summary of the white blood cell type distribution observed in mice in the pharmacodynamic experiments. Means of observed percentages are presented for each group. Percentages were not determined for mice with white blood cell counts under 1x10^3^/mm^3^.

| Immune status | Time after infection (h) | Meropenem dose group (mg/kg) | Lymphocytes (%) | Monocytes (%) | Basophils (%) | Eosinophils (%) |
| --- | --- | --- | --- | --- | --- | --- |
| Competent | 2 | 0 | 75.1 | 7.7 | 17.2 | 1.3 |
|  | 4 | 0 | 67.2 | 8.0 | 24.9 | 4.0 |
|  |  | 40 | 61.4 | 8.5 | 30.1 | 9.3 |
|  |  | 300 | 68.1 | 8.0 | 23.9 | 3.2 |
|  | 8 | 0 | 56.5 | 12.2 | 31.3 | 3.5 |
|  |  | 40 | 65.1 | 8.3 | 26.6 | 2.4 |
|  |  | 300 | 52.0 | 9.4 | 38.6 | 4.3 |
|  | 12 | 0 | 61.1 | 11.1 | 27.8 | 1.8 |
|  |  | 40 | 61.8 | 11.6 | 26.6 | 2.4 |
|  |  | 300 | 62.0 | 12.3 | 25.8 | 1.5 |
|  | 20 | 0 | 53.6 | 11.0 | 35.4 | 6.3 |
|  |  | 40 | 54.2 | 12.5 | 33.3 | 3.4 |
|  |  | 300 | 53.6 | 11.2 | 35.2 | 5.4 |
|  | 26 | 0 | 50.9 | 12.4 | 36.7 | 3.8 |
|  |  | 40 | 51.6 | 13.3 | 35.1 | 2.2 |
|  |  | 300 | 54.8 | 12.4 | 32.8 | 2.4 |
| Intermediate | 2 | 0 | 83.7 | 3.7 | 12.6 | 3.7 |
|  | 4 | 0 | 87.3 | 2.0 | 10.8 | 5.2 |
|  |  | 40 | 87.9 | 2.3 | 9.8 | 4.4 |
|  |  | 300 | 92.8 | 1.5 | 5.7 | 1.7 |
|  | 8 | 0 | 83.2 | 2.3 | 14.5 | 5.1 |
|  |  | 40 | 90.5 | 2.1 | 7.4 | 2.5 |
|  |  | 300 | 78.1 | 3.3 | 18.6 | 6.4 |
|  | 12 | 0 | 79.6 | 3.2 | 17.2 | 7.8 |
|  |  | 40 | 84.2 | 2.4 | 13.4 | 7.3 |
|  |  | 300 | 76.3 | 3.4 | 20.3 | 9.8 |
|  | 20 | 0 | 81.7 | 2.7 | 15.6 | 7.9 |
|  |  | 40 | 81.0 | 2.6 | 16.4 | 9.0 |
|  |  | 300 | 79.8 | 2.8 | 17.4 | 7.7 |
|  | 26 | 0 | 68.3 | 3.9 | 27.9 | 11.6 |
|  |  | 40 | 78.3 | 2.8 | 18.9 | 8.7 |
|  |  | 300 | 77.3 | 2.7 | 20.0 | 11.7 |
| Neutropenic | 2 | 0 | 92.6 | 2.5 | 4.9 | 3.3 |
|  | 4 | 0 | 87.7 | 2.7 | 9.6 | 6.1 |
|  |  | 40 | 86.0 | 2.1 | 12.0 | 7.1 |
|  |  | 300 | 86.0 | 2.3 | 11.8 | 7.7 |
|  | 8 | 0 | 83.9 | 2.9 | 13.2 | 7.5 |
|  |  | 40 | 84.8 | 3.3 | 11.9 | 6.4 |
|  |  | 300 | 83.4 | 3.6 | 13.0 | 6.0 |
|  | 12 | 0 | 78.4 | 3.3 | 18.3 | 9.0 |
|  |  | 40 | 77.3 | 3.0 | 19.8 | 9.8 |
|  |  | 300 | 80.4 | 4.8 | 14.8 | 11.5 |
|  | 20 | 0 | 86.1 | 2.9 | 11.1 | 4.6 |
|  |  | 40 | 92.6 | 2.0 | 5.4 | 6.1 |
|  |  | 300 | 78.7 | 3.5 | 17.8 | 9.8 |
|  | 26 | 0 | 77.6 | 3.2 | 19.3 | 10.9 |
|  |  | 40 | 75.3 | 3.5 | 21.2 | 11.6 |
|  |  | 300 | 85.4 | 2.9 | 11.7 | 5.9 |


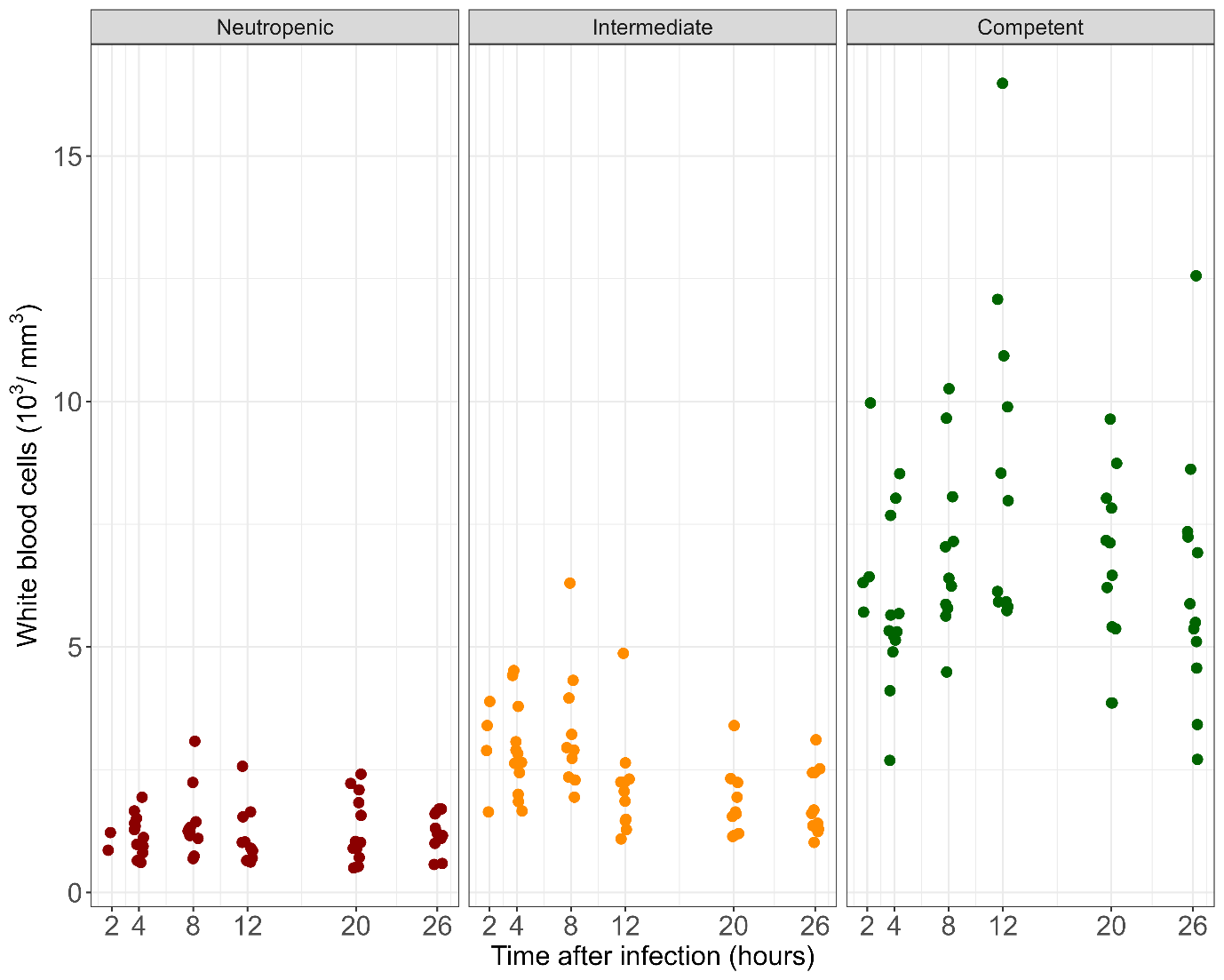


**Figure S1.** Total white blood cell counts in neutropenic, intermediate suppression, and immunocompetent mice with lung infection. Each point represents the measured white blood cell count in a single mouse. White blood cell counts were measured at the same time point as for colony-forming units (CFU) counting.


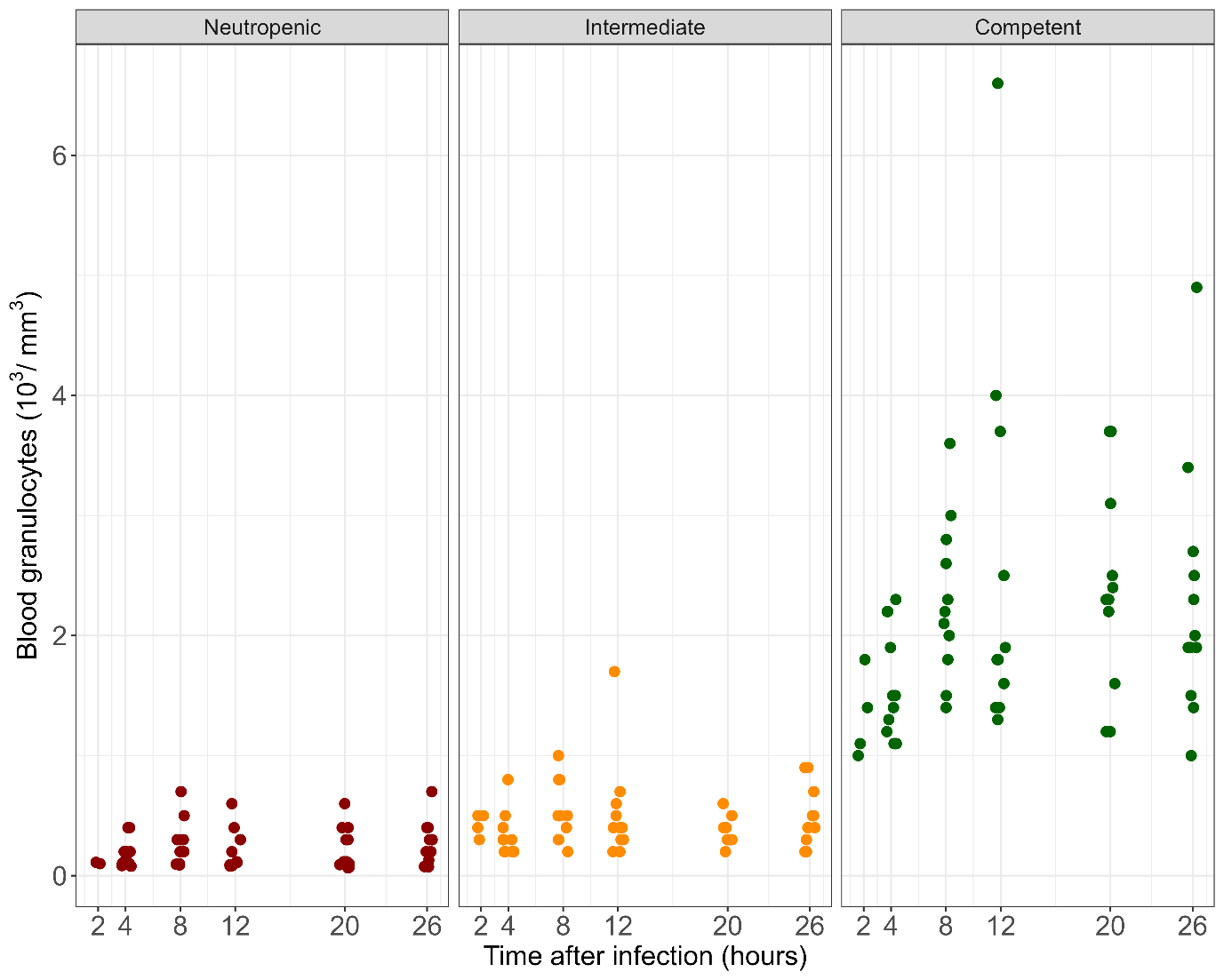


**Figure S2.** Blood granulocyte counts in neutropenic, intermediate suppression, and immunocompetent mice with lung infection. Each point represents the measured granulocyte count in a single mouse. Granulocyte counts were measured at the same time point as for colony-forming units (CFU) counting.


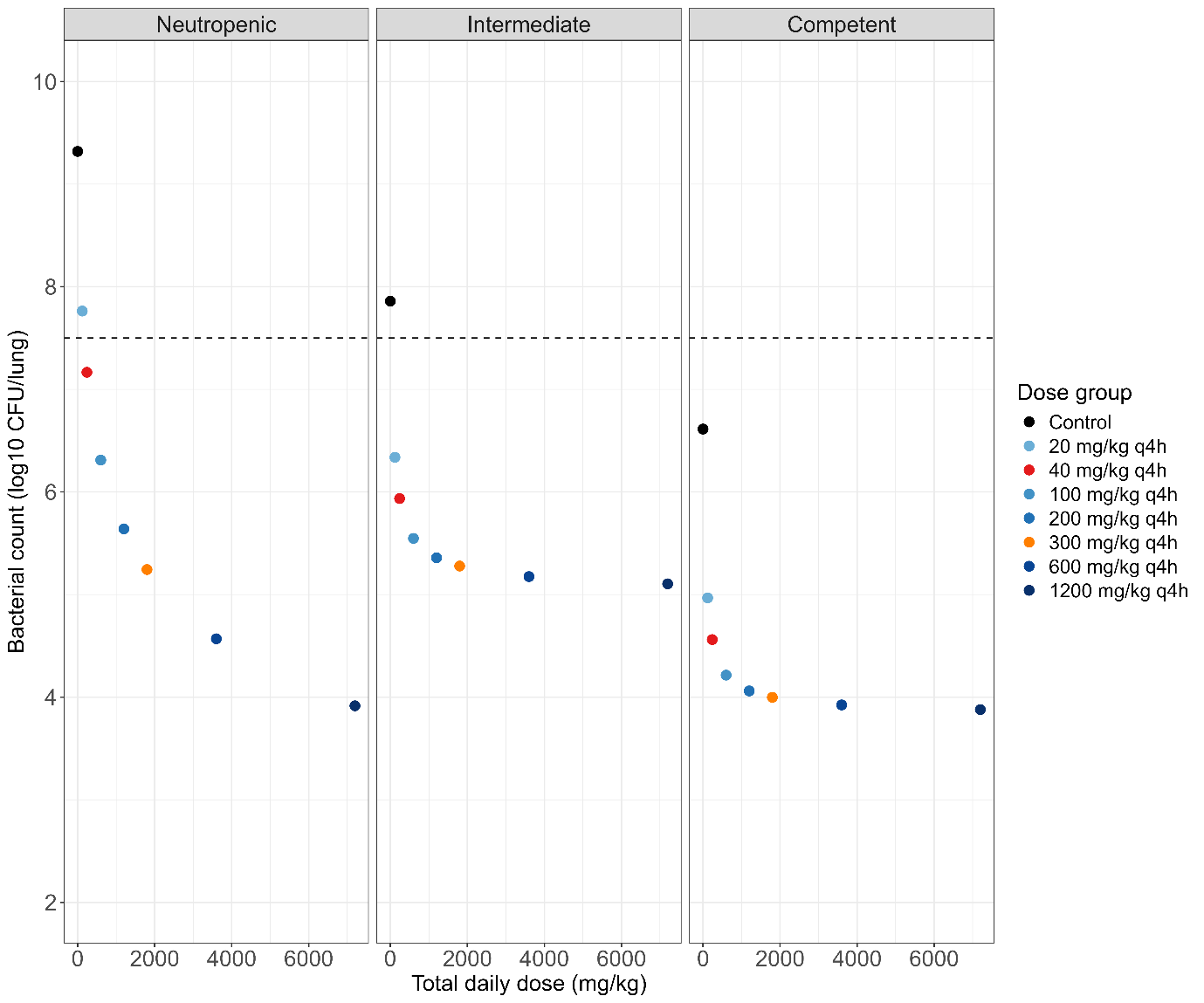


**Figure S3.** Predicted meropenem dose-response based on simulated bacterial counts at 24 hours after the start of treatment in a dose ranging study using mice in a neutropenic, intermediate suppression, or immunocompetent state. Shown are the 24-hour bacterial counts (solid circles) and the lung inoculum at start of treatment (dashed line).
